# Secure Attachment Representation in Adolescence Buffers Heart-Rate Reactivity in Response to Attachment-Related Stressors

**DOI:** 10.3389/fnhum.2022.806987

**Published:** 2022-02-17

**Authors:** Manuela Gander, Alexander Karabatsiakis, Katharina Nuderscher, Dorothee Bernheim, Cornelia Doyen-Waldecker, Anna Buchheim

**Affiliations:** ^1^Institute of Psychology, University of Innsbruck, Innsbruck, Austria; ^2^Department of Child and Adolescent Psychiatry and Psychotherapy, University of Ulm, Ulm, Germany; ^3^Department of Psychiatry, University of Ulm, Ulm, Germany

**Keywords:** stress, attachment representation, psychophysiology, adolescence, heart-rate reactivity

## Abstract

To date, we know very little about the effects of the differences in attachment classifications on the physiological correlates of stress regulation in adolescent age groups. The present study examined for the first time heart rate (HR) and heart rate variability (HRV) during an attachment interview in adolescents. HR and HRV data were collected during a baseline assessment as well as during the administration of the *Adult Attachment Projective Picture System* (*AAP*) in a community-based sample of 56 adolescents (26 females and 30 males, mean age = 16.05 years [*SD* = 1.10]). We additionally used the *Adult Attachment Interview* (*AAI*) in 50% of our sample to test the convergent validity. Adolescents with a secure attachment representation showed a higher HRV from baseline to the *AAP* interview compared to those with an insecure-dismissing (Ds) and the unresolved group. A comparison between the two insecure attachment groups showed no significant difference related to HR and HRV. Cohen’s Kappa (κ = 0.81) revealed an almost perfect agreement between the *AAP* and the *AAI* for the four-group classification. Our results indicate that adolescents with a secure attachment representation are more capable of dealing with attachment-related distress which is represented in higher HRV during an attachment interview.

## Introduction

Over the last decades literature reported a strong increase in studies focusing on the psychological, physiological, and neurobiological underpinnings of attachment (Fox and Hane, 2008; Diamond and Fagundes, 2010; Gander and Buchheim, 2015) that can influence various affective and social behaviors in humans. It is hypothesized that the attachment quality of an individual, which matures between an infant and its primary caregiver, is responsible for shaping an individual’s future relationships (i.e., romantic relationships and parent–child relationships) and has a regulatory effect on how individuals respond to attachment-related stressors. Research suggests that a secure attachment quality represents an important buffer for the physiological reactivity to stressful situations as secure individuals can balance attachment and exploration, they can use their attachment figures as a “safe haven” and they can more openly express their emotions (Diamond and Fagundes, 2010). On the other hand, insecure attachment representations are rather associated with deficits in emotion regulation. These individuals either demonstrate more feelings of anger and less autonomy (ambivalent/preoccupied) or they deactivate their attachment distress (avoidant/dismissing) that leads to a heightened physiological reactivity during attachment-related stressors (Gander and Buchheim, 2015).

Recently published studies measure autonomic imbalance during attachment-related stressful situations using indices of heart-rate variability (HRV) (Bryant and Hutanamon, 2018; Farina et al., 2015) which is considered to be important for the psychophysiological regulation of emotions in humans. HRV reflects the rhythmic oscillations of heart rate (HR) and is involved in the regulation of fight-or-flight responses. Higher HRV is associated with feeling safe and being connected to social environments as well as a greater ability to self-soothe during attachment-related stressors (Kirby et al., 2017). HR variations can be measured by a number of different methods. The most commonly used measure found in HRV research is the RMSSD (root mean square of successive differences). RMSSD is an index of short-term HRV components, thereby approximating the high-frequency domain of HRV (HF-HRV; Task Force of the European Society of Cardiology the North American Society of Pacing and Electrophysiology [TFESCNASPE], 1996). Unlike the low frequency domain, high-frequency fluctuations are known to be under predominant respiratory and parasympathetic control with only negligible sympathetic contributions, as validated by work using pharmacological blockades, among others (Berntson et al., 1993). As RMSSD seems to be less affected by fluctuations in respiration, researchers often consider RMSSD an index of parasympathetic activity or vagal control (e.g., Hill et al., 2009; Wulsin et al., 2018). In several studies increases in HF-HRV, which are indicative of vagal upregulation, have been linked to positive social engagement and better coping strategies in response to stressors (Porges, 2011; Hastings and Miller, 2014; Bornemann et al., 2016). Furthermore, studies have demonstrated that increases in HF-HRV are associated with altruistic feelings and behaviors. In this regard, the HF-HRV upsurge during altruistic actions might reflect an individual’s deliberate self-regulatory effort and activate the emotional-motivational systems related to caregiving and positive social engagement (Bornemann et al., 2016).

Concerning attachment-related situations, higher HF-HRV is associated with feeling safe and being connected to social environments as well as a greater ability to self-soothe during attachment-related stressors (Kirby et al., 2017). Studies using the *Strange Situation Procedure* (Ainsworth, 1979) demonstrated that securely attached infants show acceleratory trends of their HR, the number of heartbeats per time interval, after separation (Donovan and Leavitt, 1985), and recovery within less than a minute after reunion with their attachment figure (Sroufe and Waters, 1977). Furthermore, the HR declines when the unfamiliar adult approaches them (Donovan and Leavitt, 1985) and there is a concordance between the maternal and the infant HR (Zelenko et al., 2005; Feldman et al., 2011). These results indicated that securely-attached infants can adequately cope with attachment-related negative information during emotional processing. They might also have a better ability to orient to a stranger for comfort and their primary caregivers might be involved in their behavior (Gander and Buchheim, 2015).

Studies on cardiovascular stress responses among the different attachment representations in adults are based on two widely used attachment interviews, namely the *Adult Attachment Interview* (*AAI*; George et al., 1996) and the *Adult Attachment Projective Picture System* (*AAP*; George and West, 2001; George and West, 2012). The *AAI* (George et al., 1996) was developed to assess the four main attachment representations based on the evaluation of attachment-related autobiographical memories from early childhood. The *AAP* (George and West, 2001) is the second valid narrative attachment measurement that extends the projective methodology employed in child attachment research. It consists of line drawings that depict relevant attachment scenes of illness, separation, or death. Individuals are asked to tell a short story about each picture stimuli. In line with the *AAI* coding, the *AAP* classification system also designates four attachment patterns [secure, insecure-dismissing (Ds), insecure-preoccupied, and unresolved (George and West, 2012)].

Individuals with a secure attachment (F) are characterized by experiences of emotional support, comfort, and the availability of their caregivers during stressful situations. Thus, they feel competent and valuable. In contrast, Ds and insecure-preoccupied (E) individuals experienced rejection or inconsistent responses from their caregivers in situations when they needed them. As a consequence, they tend to devaluate or idealize their attachment experiences (deactivating attachment distress in case of Ds) or they show anger and low autonomy as they are enmeshed with their caregivers (hyperactivating attachment distress in case of E). Unresolved individuals (U) experienced their caregiver’s failure to soothe their hyperarousal and restore safety in situations of severe attachment distress, leaving them in a psychological state of isolation and feeling helpless.

The *AAP* has been used successfully in neurobiological attachment research in adults (Buchheim et al., 2006; Stanley, 2006; Fraedrich et al., 2010). However, so far most studies on cardiovascular stress responses measuring physiological correlates of human attachment are based on the use of the *AAI* (Dozier and Kobak, 1992; Beijersbergen et al., 2008; Rifkin-Graboi, 2008). On a physiological level, adults with a secure-autonomous attachment demonstrated a lower increase in cardiovascular reactivity from baseline to questions during the *AAI* related to a threat, rejection, and separation compared to the insecure groups (Dozier and Kobak, 1992; Roisman et al., 2004; Roisman et al., 2007; Holland and Roisman, 2010). Farina et al. (2015) focused on the unresolved attachment group in adult patients with dissociative disorder. This attachment group is characterized by a breakdown of defensive and coping strategies in response to stressful attachment situations which might be reflected in higher physiological arousal during attachment-related stressors compared to the other three attachment groups. The authors found that patients with an unresolved status (*n* = 13) showed a change in the heart-rate pattern characterized by a significant increase in the low frequency (LF)/high frequency (HF) ratio after the *AAI* (Farina et al., 2015) suggesting these variables being psychophysiological correlates of emotion dysregulation in these patients.

Furthermore, some studies classified attachment representations using the *AAI* or the *AAP.* They observed cardiovascular reactivity in other situations where adults activate their attachment system (Feeney and Cassidy, 2003) using marital-conflict tasks or rejection experiences. Results revealed a lower cardiovascular reactivity in secure adults during a conflict-interaction task. This finding indicated that they rather share their thoughts and opinions with their marital partners when discussing an issue of disagreement than those with an insecure attachment pattern (Roisman, 2007). Comparable results were found by De Rubeis et al. (2016). Using the *AAP* they reported significantly lower increases in HR during a rejection experience (Cyberball experiment) in depressed individuals with a resolved attachment pattern (F, Ds, and E) than in unresolved individuals using the *AAP*. Yet more research is needed before final conclusions can be drawn regarding the specific cardiovascular responses in individuals with different attachment patterns.

The study of neurophysiological correlates of attachment in groups of adolescent age is still a relatively untapped area of research (Gander and Buchheim, 2015). In the only study to date available using attachment interviews, Beijersbergen et al. (2008) examined the cardiovascular response during the *AAI* and a family-interaction task. In contrast to findings in infants (Sroufe and Waters, 1977; Donovan and Leavitt, 1985) and adults (Dozier and Kobak, 1992; Roisman et al., 2007), the authors did not observe any significant differences in HRV between secure and Ds adolescents in response to the *AAI*. However, during the conflict-interaction task with their mothers, the Ds group demonstrated an elevated HR. This rather unexpected finding might suggest that adolescents with an Ds attachment representation are probably less open to the *AAI* procedure and thus deal with questions on childhood experiences in a more superficial manner than adults (Beijersbergen et al., 2008). There might also be developmental issues specific to groups of adolescent age that could be relevant in interpreting this finding. One profound transformation regarding attachment in adolescence is to achieve independence from parents. By establishing a critical distance to their parents, young people start to objectively re-evaluate the nature of their attachment relationships (Kobak and Cole, 1994; Allen, 2008). This striving for autonomy is often considered as a crucial factor for the higher percentage of Ds attachment classifications that researchers found in adolescents compared to adult cohorts (Bakermans-Kranenburg and van IJzendoorn, 2009; Gander et al., 2016).

Taken together, the literature suggests that a secure attachment representation serves as an important buffer in response to attachment-related stressors that can also be evidenced by a higher HRV and weaker increase in HR. However, there is a paucity of research on adolescents. The only study conducted in adolescents unexpectedly failed to confirm the cardiovascular characteristics found in infants and adults during attachment measurements. These contradictory findings raise the central question of whether results on adolescent attachment security and physiological functioning are different from those reported in childhood and adulthood (Beijersbergen et al., 2008).

To further fill this gap of knowledge, the present study examined HR and HRV during the *AAP* interview in adolescents. To expand the research results from the only existing study in this field (Beijersbergen et al., 2008), we assessed a representative community sample of adolescents who were primarily raised by their biological parents. Furthermore, we covered a wider developmental period ranging from 14 to 18 years of age and included a broader variety of sociodemographic characteristics, especially regarding family issues (i.e., marital status of parents, household situation, etc.). As we used a different attachment interview than the study of Beijersbergen et al. (2008), we additionally assessed the *AAI* in more than half of our sample to demonstrate convergent validity for these two instruments in adolescence. Concerning the physiological correlates we tested the following hypothesis: (1) we hypothesized that secure adolescents show an increased HRR (HR and HRV) from the neutral baseline to the *AAP* interview; (2) we assumed that adolescents with an insecure attachment representation and in particular those with an unresolved attachment pattern demonstrate a lower HRV from the baseline to the *AAP* picture stimuli because they are less capable to self-soothe and they feel less safe and connected and thus experience more physiological stress during the *AAP* than those with a secure attachment representation.

## Materials and Methods

### Sample

The initial sample consisted of *N* = 87 adolescents between 14 and 18 years of age. They were recruited in different areas of Southern Germany and Austria between 2015 and 2017 by shared flyers, email, and public advertisement. We included adolescents with sufficient knowledge of the German language and normal body weight (body mass index [BMI] between 18.5 and 24.9). The present study was approved by the institutional review board (IRB) of the University of Innsbruck and we obtained written informed consent from both the adolescents and their parents for study participation. Due to technical difficulties resulting in incomplete hardware data recordings during the physiological measurements, datasets from 23 adolescents were excluded from the statistical analyses. Our final sample consisted of 56 adolescents (26 females and 30 males) with a mean age of 16.05 years (*SD* = 1.10). Characteristics of the study cohort are given in Table 1.

**TABLE 1:** Sociodemographic characteristics among the four attachment groups.

Total (*N* = 56)	*F*		Ds		*E*		*U*		Φ		*p*	
	*n* = 19		*n* = 24		*n* = 7		*n* = 6					
**Sex (%)**												
Male	63.2		41.7		42.9		83.3		0.28		0.210	
Female	36.8		58.3		57.1		16.7					
**Living situation (%)**												
Living alone/foster care	0		8.3		0		33.3		0.38		0.041	
Living with parents	100		91.7		100		66.7					
**Amount of siblings (%)**												
Single child	5.3		0		0		0		0.34		0.687	
One sibling	47.4		50.0		57.1		33.3					
Two siblings	26.3		41.7		14.3		33.3					
More than two siblings	21.1		8.3		28.6		33.3					
**Marital status of parents**												
Married/partnership	73.7		83.3		42.9		66.7		0.36		0.317	
Single/divorced	21.1		16.7		57.1		33.3					
Deceased	5.3		0		0		0					
**Occupation**												
Attending school	94.7		95.8		100.0		100.0		0.24		0.770	
Employed/trainee	0		4.2		0		0					
Unemployed	5.3		0		0		0					

	***M***	***SD***	***M***	***SD***	***M***	***SD***	***M***	***SD***	***F***	***df***		***p***

Age	16.26	1.05	15.96	1.20	15.57	0.98	16.33	1.03	0.857	3		0.469

*F, secure; Ds, insecure-dismissing; E, insecure-preoccupied; U, unresolved.*

### Attachment Measurements

*The AAP* (George and West, 2012), measures the four attachment patterns F, Ds, E, and U based on a set of eight picture stimuli that include one neutral and seven attachment-related line drawings depicting scenes of separation, death, illness, or solitude. Adolescents are asked to tell a short story about each picture using the following standardized questions (George and West, 2012): “What led up to that scene? What are the characters thinking or feeling and what might happen next”? Based on verbatim transcripts, each picture stimulus is coded for content (“haven of safety,” “internalized secure base,” and “synchrony”) and defensive processes (deactivation, cognitive disconnection, and segregated systems). For a more detailed description of these markers and the coding procedure see Table 2 (George and West, 2011, 2012). Several studies demonstrated convincing psychometric properties of the *AAP* in adults (Buchheim et al., 2008; Benoit et al., 2010; George and West, 2012) and adolescents (Gander et al., 2016) by showing an almost perfect inter-rater reliability [concordance rate was 90%, κ = 0.85, *p* < 0.001 see George and West (2001); Buchheim et al. (2008); Benoit et al. (2010)], discriminant validity in controls and clinical patients (George and West, 2001, 2012) and test–retest reliability (0.84 across 3 months). Furthermore, there is evidence for good concurrent validity with the *AAI* in adult samples [concordance rates for the four-group attachment classifications (F, Ds, E, and U) were 90%, κ = 0.84, *p* < 0.001 and for the two group attachment classification (secure vs. insecure) even 97%, κ = 0.89, *p* < 0.001] (Buchheim and George, 2011; George and West, 2012). A recent study on adult patients with chronic depression and non-depressed controls demonstrated high convergent validity between the *AAI* and the *AAP* in *N* = 30 participants, κ = 0.89 (ASE = 0.112), *p* < 0.001, simple agreement 94% (Buchheim et al., 2018).

**TABLE 2:** Overview on the defenses assessed with the *Adult Attachment Projective Picture System* (AAP).

AAP defenses	Definition	Language markers
Deactivation	Deactivate, diminish or devalue themes to produce a representational distance between the individual and the attachment-related event	Power, achievement, authority, distance, rejection, neutralization, and social rules
Cognitive disconnection	A mental state characterized by confusion, mental shifts and opposite descriptions of attachment events and emotions.	Uncertainty, heightened emotional arousal, anger, withdrawal, opposed images or choices
Segregated systems	A mental state in which painful attachment-related experiences are isolated and blocked from the consciousness leaving the individual in a momentary or prolonged state of attachment dysregulation	Helplessness, isolation, frightened, out of control, abandoned, one’s own traumatic experience, failed protection

However, one study failed to find a sufficient convergent validity between the *AAP* and the *AAI* in adult students attending a public university in the United States (Jones-Mason et al., 2015) suggesting that these two measurements probably do not assess the same construct of attachment in younger cohorts. Unfortunately, no study tested the convergent validity in adolescents between 14 and 18 years of age, and thus this conclusion needs further support from empirical evidence. Consequently, we decided to use the *AAI* in more than half of our adolescent sample (*n* = 32) to additionally co- investigate for the first time the concurrent validity between these two instruments for a younger age group.

*The AAI* (George et al., 1996; Hesse, 2008) is the most widely used clinical-style interview and is often referred to as the “Gold standard” for the assessment of attachment representation. In the last three decades, the *AAI* has also extensively been used in adolescent populations (Bakermans-Kranenburg and van IJzendoorn, 2009). During the interview procedure, subjects are asked about various memories of their childhood relationships with their primary caregivers, including frightening events of abuse and loss. It taps their evaluations of these experiences and how they have impacted their adult personality. The verbatim transcripts are coded for inconsistencies and incoherence of the discourse. As previously stated, the *AAI* also classifies the four different attachment groups F, Ds, E, and U (for more information on the *AAI* coding procedure see Bakermans-Kranenburg and Van IJzendoorn, 1993; George et al., 1996; Hesse, 2008; Ravitz et al., 2010). In terms of psychometric properties, the *AAI* has demonstrated good construct validity (George et al., 1996), test–retest reliability and discriminant validity (Bakermans-Kranenburg and Van IJzendoorn, 1993; Crowell et al., 1996) and satisfactory inter-rater reliability for the four group classifications, κ = 0.77, *p* < 0.001 (Jones-Mason et al., 2015).

### Psychological Measures to Assess Psychological Distress

*The Brief Symptom Checklist* (*BSCL*) was used to measure our adolescents’ overall psychological distress level at the time of the assessment procedure. We also used the *Global Severity Index* (*GSI*; Derogatis and Melisaratos, 1983) which was designed to help to quantify an individual’s symptom severity on a single composite score. The *BSCL* provides norms for adolescent populations (Derogatis and Melisaratos, 1983; Geisheim et al., 2002).

### Physiological Measurements for Heart Rate and Heart-Rate Variability

The biofeedback-measurement system (NeXus-10 MkII, Mind Media AG, Germany) used in this study is a multimedia data-acquisition tool for measuring a variety of physiological signals simultaneously.

Heart rate reactivity data was collected by an electrocardiogram (ECG). Three electrodes were placed onto the adolescent’s chest: the first two under the left and right collarbones, and the third under the left lower ribs. Before the physiological measurement, we asked the adolescent about a possible history of myocardial diseases (*N* = 0), current medication intake (*n* = 10 took contraceptives at the time of the assessment), and cigarette smoking (*n* = 7). During the *AAP*, the HRR of the adolescent participants were recorded. The parameter bpm (beats per minute) measures the number of heartbeats per minute recorded in real-time. According to the American Heart Association, the physiological HR ranges between 60 and 100 bpm. We used a 250-Hz sampling frequency for our HRV analysis. We measured HRV which is divided into two parameter subsets: the RMSSD and the SDNN (standard deviation of NN intervals), both measured in milliseconds (ms). These values show the changes in the time interval between successive heartbeats and the variable reaction to outer or inner influences (Hottenrott, 2002). As SDNN represents an estimate of total HRV which encompasses high and low-frequency as well as very and ultra-low frequency domains, it is considered an imprecise index which cannot distinguish between effects of the sympathetic and the parasympathetic branches of the autonomic nervous system (Berntson et al., 1997). Therefore, we did not include results on SDNN in the present study. As artifacts can contribute to substantial spurious broadband power in estimates of heart period variability (Berntson and Stowell, 1998) we analyzed the raw digitized ECG signals to identify possible outliers in the physiological data (>1.5 interquartile ranges) and then processed them by BioTrace+ software for measures of HR and heart rate variability (HRV). Our device features flawless signals that are minimized by True Active Shielding (TAS) technology and additional carbon coated cables for minimal artifacts and noise. The BioTrace+ software was used for physiological monitoring, data analysis and to transfer data to SPSS. The BioTrace+ software allows to measure the individual setting of the channels. The scale rages from 32 SPS-8192 SPS. Thus, the ECG signal was measured with a SPS of 4096 which speaks for a high measurement accuracy.

### Procedure

**FIGURE 1:**
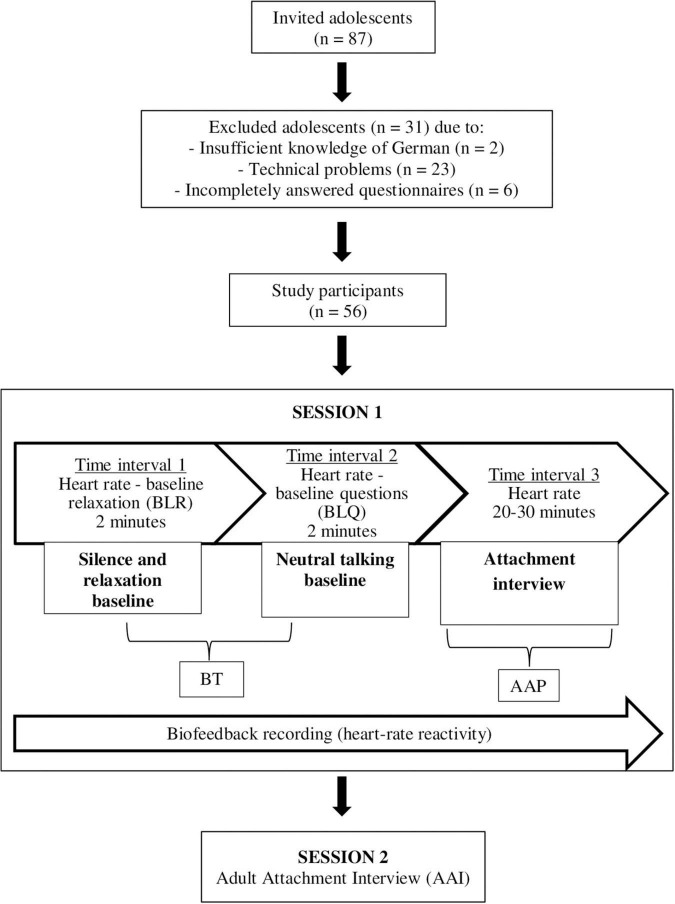
Participant’s exclusion and inclusion criteria and graphical representation of the experimental workflow.

Adolescents and their parents gave their written informed consent after receiving a detailed description of the study and assurance of the pseudonymity of their data. Next, adolescents (without their parents) were scheduled for a first assessment at the research laboratory where they completed the *AAP* interview while their physiological data was recorded. To avoid daily influence on motivation and mental condition we used only measurements taken during the afternoon between 2 and 4 p.m. First, the participants were connected to the biofeedback system to record the entire session. They were asked to sit down on a chair and adopt an upright and relaxed position. The measurement recording started after preparing the participants and checking the quality of the recording signals at the beginning of the experimental procedure. A graphical representation of the test sequence is shown in Figure 1.

The first parameter termed *Baseline relaxation* (*BLR*) was calculated from the mean signal recorded over a 2-min time interval during environmental silence and relaxation phase of the participant. Next, the subjects answered nine neutral questions to build the second parameter *Baseline questions* (BLQ). Using this method allowed us to control for a potential influence of verbal speech. The BLQ only represented a pilot trial. It was used to generate the parameter *Baseline total* (BT) in combination with the BLR. In the next step, the standard instruction of the *AAP* was applied and the voice recording started.

After completing the *AAP*, the *AAI* interview was administered in a second session with *n* = 32 participants. Each adolescent received 50 € as a compensation fee for study participation. Six students of psychology trained for all laboratory procedures conducted the *AAP* and recorded the physiological measurements under the supervision of a certified *AAP* rater with the same technical expertise. The *AAI* interview was conducted by two other students who also received extensive training from academic staff. The coding of the verbatim *AAP* and *AAI* transcripts were done by two independent, licensed judges.

### Data Analysis and Statistics

Statistical analyses were performed with IBM SPSS statistical software for Windows (version 25.0). Normal distribution of data and residuals was tested using the Kolmogorov–Smirnov test and Shapiro–Wilk test, respectively. First, we calculated the distribution of the attachment classifications in our cohort. We examined differences in sociodemographic variables using Chi-square tests (sex, occupation, marital status of the parents, and amount of siblings) and ANOVA (age) followed by *post hoc* Bonferroni corrections. Second, we established inter-rater reliability for the *AAP* and convergent validity for the *AAI* as well as the *AAP* coding using Cohen’s kappa and Chi-square tests. Next, we calculated the HRR difference between the two baselines (BLR and BLQ) and the *AAP classifications*. These variables were formed by subtraction of the average arousal level during the *AAP* and the mean values of the two baselines calculated for BT and BLR. A Wilcoxon test was conducted to test the non-parametric RMSSD between groups across experimental conditions. Differences in mean baseline values between the four different *AAP* groups were calculated using the Mann–Whitney *U*-Test. Based on our hypotheses derived from previous research results we did a two-step correction: First, we corrected for type 1 error, followed by a Benjamin–Hochberg correction. A level of significance of *p* < 0.001 was applied to our significance testing. Furthermore, we used Cohen’s (1998) conventions for effect sizes: *d* = 0.2 is considered as a small effect, *d* = 0.5 is a medium effect and *d* = 0.8 is a large effect.

### Characteristics of the Study Cohort

The distribution of attachment classifications by *AAP* in our sample of adolescents was as follows: We classified *n* = 19 participants as “secure” (34%), *n* = 24 as “Ds” (43%), *n* = 7 as “insecure-preoccupied” (13%) and *n* = 6 as “unresolved” (11%). In line with the study of Gander et al. (2016) our results did not show any significant relation to sociodemographic variables like (a) sex χ^2^ (3, *n* = 56) = 4.529, *p* = 0.210, Φ = 0.28, (b) occupation χ^2^ (6, *n* = 56) = 3.304, *p* = 0.770, Φ = 0.24, (c) marital status of the parents χ^2^ (6, *n* = 56) = 7.004, *p* = 0.317, Φ = 0.36, (d) the amount of siblings χ^2^ (9, *n* = 56) = 6.515, *p* = 0.687, Φ = 0.34 and (e) age *F*(3,52) = 0.86, *p* = 0.469, $\eta_{\text{p}}^{2}$ = 0.047, *d* = 0.44. However, in the unresolved group significantly more adolescents lived in foster care χ^2^ (3, *n* = 56) = 8.256, *p* = 0.041, Φ = 0.38. The summary of this data is shown in Table 1. Furthermore, we found that adolescents with a secure attachment pattern reported significantly lower psychological distress (*M* = 17.79, *SD* = 15.21, *Mdn*: 22.79) on the *Global Severity Index* of the *BSCL* than the two insecure groups (*M* = 28.19, *SD* = 26.31, *Mdn*: 29.34) and the unresolved group (*M* = 43.83, *SD* = 27.51, *Mdn*: 42.25), χ^2^ (2, *n* = 56) = 6.682, *p* = 0.035.

## Results

### Inter-Rater Reliability and Concurrent Validity Between *Adult Attachment Interview* and *Adult Attachment Projective Picture System*

Two independent certified judges coded the *AAP* transcripts. Inter-rater reliability analyses revealed a kappa for the four-group classification of 98%, κ = 0.974 with a narrow 95% confidence interval [0.923, 1.025], *p* < 0.001. The two independent raters agreed in as many as *N* = 55 out of *N* = 56 cases showing a very high concordance between the two judges. The disagreement between the judges for the one case was resolved after direct inter-rater communication.

In a second step, we analyzed the convergent validity between the *AAP* and the *AAI* attachment classifications coded by one independent certified judge in our adolescent sample (*n* = 32). Our analysis shows a almost perfect agreement between the *AAP* and the *AAI* classifications (94%, κ = 0.89 with a 95% narrow confidence interval [0.680, 1.100], *p* < 0.001) for the two group classifications (resolved-unresolved) and 81%, κ = 0.81 with a 95% narrow confidence interval [0.641, 0.982], *p* < 0.001 for the four-group classifications (F, Ds, E, and U). Table 3 demonstrates the frequencies of the *AAP* and the *AAI* attachment classifications.

**TABLE 3:** Attachment-classification overlap between the *AAP* and the *AAI*.

	AAI classification				
AAP classification	*F*	Ds	*E*	*U*	Total
F	9	0	0	0	9
Ds	1	13	0	0	14
E	0	2	1	0	3
U	0	1	0	5	6
Total	10	16	1	5	32

*F, secure; Ds, insecure-dismissing; E, insecure-preoccupied; U, unresolved. p < 0.001, Kappa = 0.79.*

### Differences in Heart-Rate Reactivity Between the Four Attachment Groups

A Kruskal–Wallis test revealed significant differences in mean score change from baseline to the *AAP* among all attachment groups for RMSSD, χ^2^ (3, *n* = 56) = 8.963, *p* = 0.03. Our results further showed a significant increase in HR [(*M* = 82.9, *SD* = 13.65), *t*(55) = −2.38, *p* = 0.021, *d* = 0.32] across all attachment groups. A Mann–Whitney *U* tests for pairwise comparisons showed a higher RMSSD in individuals with a secure attachment pattern (F) compared to individuals with an Ds (RMSSD: *U* = 126, *p* = 0.013, *r* = 0.38) and an unresolved attachment pattern (RMSSD: *U* = 23, *p* = 0.031, *r* = 0.43). *Post hoc* comparisons using a Benjamin–Hochberg correction indicated that these differences remain statistically significant between the secure and the Ds group (RMSSD: *p* = 0.019, 90% CI [1.594, 26.197]) and the unresolved group (RMSSD: *p* = 0.018, 90% CI [1.655, 26,137]). The analysis between the two insecure attachment groups Ds and E showed no significant physiological difference in HRV (RMSSD: *U* = 58, *p* = 0.741, *r* = 0.03) (see Table 4). We could not find a statistical difference for HR from total baseline procedure to the *AAP* among the four attachment groups.

**TABLE 4:** Means and standard deviations for physiological parameters among the four attachment groups from the total baseline to the attachment interview.

	Min_diff_ (*n* = 56)	Max_diff_ (*n* = 56)	*F* (*n* = 19)			Ds (*n* = 24)			*E* (*n* = 7)			*U* (*n* = 6)			*F* vs. Ds			*F* vs. *E*			*F* vs. *U*		
			*M*_diff_	*SD*_diff_	*Mdn*	*M*_diff_	*SD*_diff_	*Mdn*	*M*_diff_	*SD*_diff_	*Mdn*	*M*_diff_	*SD*_diff_	*Mdn*	*U*	*r*	*p*	*U*	*r*	*P*	*U*	*r*	*p*
RMSSD	−40.45	61.80	11.5	19.36	27.37	−2.4	12.75	17.57	8.0	6.74	11.86	3.1	15.64	7.33	126	0.38	0.013	55	0.13	0.534	23	0.43	0.031
HR	−7.45	13.38	1.3	5.50	21.79	1.7	4.62	22.17	0.9	5.60	12.57	1.6	4.87	14.08	224	0.02	0.922	60	0.07	0.735	50	0.08	0.687

*AAP, Adult Attachment Projective Picture System; RMSSD, root mean square of successive differences; F, secure; Ds, insecure-dismissing; E, insecure-preoccupied; U, unresolved; M_diff_, mean value of the difference score from baseline to the AAP interview; SD_diff_, standard deviation of the difference score from the baseline to the AAP interview; Mdn, median.*

### Heart Rate Reactivity in the Secure and the Insecure (Insecure-Preoccupied, Insecure-Dismissing, and Unresolved) Group

**FIGURE 2:**
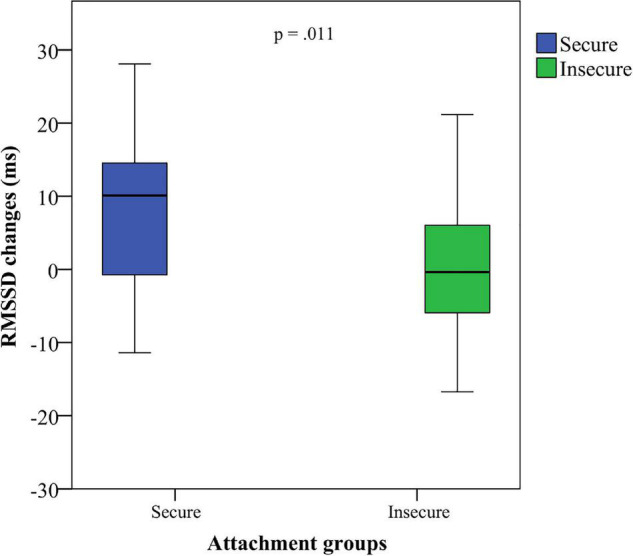
RMSSD changes of adolescents classified as secure and insecure from the total baseline to the attachment interview.

**FIGURE 3:**
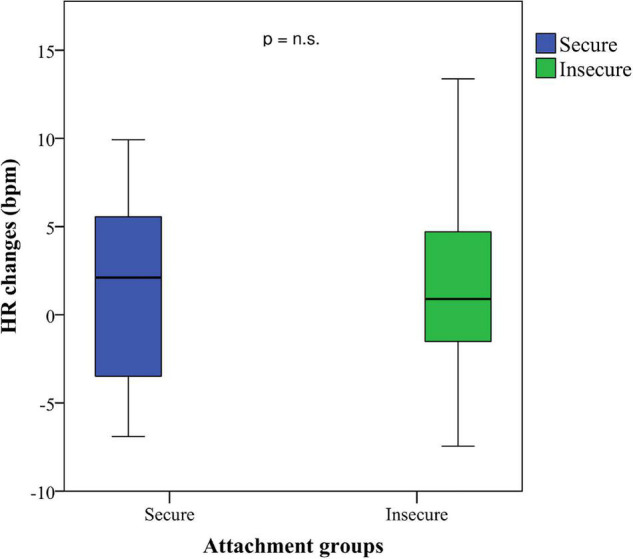
HR changes of adolescents classified as secure and insecure from the total baseline to the attachment interview.

**FIGURE 4:**
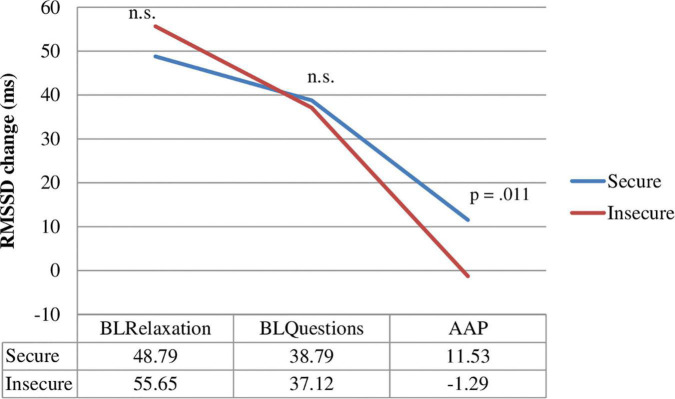
RMSSD measurement of adolescents classified as secure and insecure for the baseline relaxation (BLR), the baseline questions (BLQ), and the attachment interview (AAP).

A Mann–Whitney *U*-test showed a significant difference for RMSSD between secure adolescents (*M* = 6.49, *SD* = 24.92, Mdn: 35.37) and insecure adolescents (*M* = −6.18, *SD* = 14.07, Mdn: 24.97), *U* = 221, *p* = 0.024, *r* = 0.30, from the BLR to the *AAP*. These results remain significant for the total baseline procedure (BT) to the *AAP* (RMSSD: secure *M* = 11.53, *SD* = 19.36, Mdn: 36.26; insecure *M* = −1.29, *SD* = 11.36, Mdn: 24,51), *U* = 204, *p* = 0.011, *r* = 0.34, suggesting that secure participants show a higher HRV during the interview procedure (see Figures 2–4). This difference could not be found for HR between the secure and the insecure group.

## Discussion

Our study investigated whether attachment representations are related to heart-rate reactivity during an attachment interview (*AAP*) in adolescents between 14 and 18 years of age. We classified attachment groups using the *AAP* and the *AAI* and found that the majority of our adolescent cohort had a secure or an insecure dismissing attachment pattern. On a physiological level, the lower HRV (baseline compared to *AAP* picture stimulation) in Ds and unresolved individuals suggests that they experience more physiological stress during the attachment interview than those with a secure attachment pattern. However, we did not find significant differences in HRV between secure and insecure-preoccupied adolescents.

In our sample, the majority of individuals were classified with a secure or an Ds attachment representation in the *AAP* and the *AAI*. These results are consistent with findings from studies using the *AAP* or the *AAI* in teenagers (Bakermans-Kranenburg and van IJzendoorn, 2009; Gander et al., 2016). Furthermore, we found a very good convergent validity between *AAP* and *AAI* classifications for our adolescent sample. Although there is one study in young adults that found a divergent result (Jones-Mason et al., 2015), our finding is in line with the majority of other studies that reported a good agreement for groups of adult age (Buchheim et al., 2008; Buchheim and George, 2011; George and West, 2011; George and West, 2012; Buchheim et al., 2018).

The major finding of this study was that adolescents with a secure attachment representation showed a higher HRV from baseline to the *AAP* compared to adolescents with an insecure attachment representation. Our results are in line with several studies that found lower HRV levels in individuals with an insecure attachment pattern during laboratory stress exposition (Maunder et al., 2006; Diamond and Fagundes, 2010). The more reactive HRV of secure adolescents suggests that they are more capable of dealing with attachment-related distress during the *AAP*. In this context, interdisciplinary research approaches should be used to co-assess biological signaling molecules of attachment, e.g., biofluid levels of oxytocin (Krause et al., 2016) or prolactin which should be co-assessed in future studies.

Previous research demonstrated that individuals who were classified as “secure” showed an ability to think about attachment-related stressors constructively, they could reach out for attachment figures for comfort and safety under conditions of danger, and their attachment relationships were characterized by mutual enjoyment and responsiveness (George and West, 2011, 2012). Even though they sometimes use defensive strategies when they are highly stressed (i.e., by distancing themselves from stressful situations) this serves as a way to flexibly integrate attachment-related feelings and events instead of excluding them from the consciousness as they are too painful or overwhelming (Bowlby, 1969; George and West, 2011). These adaptive ways of coping with negative emotions develop and grow most effectively in the context of secure attachment relationships in childhood (Allen and Miga, 2010). The greater increase in RMSSD observed in secure individuals might reflect more adaptive coping strategies in response to stress which is also evidenced in some of the aforementioned studies establishing links between deliberate vagal upregulation and social engagement (Porges, 2011; Hastings and Miller, 2014; Bornemann et al., 2016). Similarly, researchers found a more pronounced parasympathetic activity during a social exclusion task (Cyberball) as indexed by vagally mediated event-related transient cardiac slowing in a large longitudinal study of pre- and school-age children with greater positive parent representations (White et al., 2021). This data suggests that children with a secure attachment might have a moderately sensitive and receptive alarm system that assists them to attend more closely to negative social stimuli in order to foster adaptive responses accounting for more peer competence and fewer peer problems.

On the other hand, the lower HRV levels of Ds adolescents indicate a greater stress level and lower resiliency to attachment-related stressors. One reason could be that they demonstrate many deactivating defensive strategies that maintain a distance in relationships by describing characters as very autonomous and including themes of achievement and exploration in their narratives (George and West, 2012). These defensive mechanisms help dismissing individuals to suppress the direct expression of attachment feelings, memories, and behaviors to emphasize their self-sufficient strength and independence that does not require attachment figures for safety and comfort (George and West, 2012). Even though these individuals adaptively manage to maintain closeness to their caregivers by removing distressing attachment feelings from the consciousness, they do not completely manage to suppress their negative feelings of anger, sadness and anxiety in attachment relationships that becomes particularly apparent in their heightened physiological arousal during attachment-related stressors (Dozier and Kobak, 1992; Diamond et al., 2006; Ablow et al., 2013). Research on adolescent psychopathology also suggests that avoidant coping did not help deal with an overload of stress and thus contributes to the longitudinal prediction of symptomatology (Seiffge-Krenke, 2000).

Similar to Ds adolescents, the group “unresolved” also demonstrated significantly lower RMSSD from the baseline to the *AAP* interview than “secure” adolescents. This finding suggests that unresolved individuals are not only exposed to a high physiological stress level during the interview procedure, but they also seem to be more challenged to adapt constructively to the various forms of attachment-related distress and trauma evoked by the *AAP* stimuli. Our results are similar to findings from infant studies during the *Strange Situation Procedure* (Spangler and Grossmann, 1993; Willemsen-Swinkels et al., 2000) that demonstrated a higher HR in disorganized infants when their attachment figures were present in the same room. Even though several studies linked moderate vagal withdrawal indicated by reductions in HF-HRV to more effective emotion regulation (Katz and Rigterink, 2012) and meta-analytically fewer behavioral problems (Graziano and Derefinko, 2013), these positive effects of vagal withdrawal are primarily detected for non-social stressful contexts. However, in social contexts, adaptive self-regulatory behavior may well be supported by vagal upregulation (Bornemann et al., 2016). In our study, the AAP may have lead individuals with a secure attachment representation to deliberately upregulate their vagal activity to more effectively engage with the social content and the interpersonal interview situation.

Yet interestingly, we did not find significant differences for HRV between the secure and the insecure-preoccupied group. The study of Beijersbergen et al. (2008) also found no significant difference in RMSSD between these two groups during the *AAI*. In contrast to Ds or unresolved individuals, those with an insecure-preoccupied attachment representation employ maximizing strategies to activate their caregivers’ responses through exaggerating distress cues and focusing excessively on attachment-related situations (Cassidy and Berlin, 1994). It can be hypothesized that these strategies might be more adaptive in gaining a caregiver’s attention than those of Ds or unresolved individuals. This might result in HRV patterns that are similar to those of secure individuals (Cassidy and Berlin, 1994; Beijersbergen et al., 2008). Other studies also found evidence for a more adaptive stress regulation in those with an insecure-preoccupied attachment pattern. For example Borelli et al. (2021) found similar results in 8–12 years old children with an insecure-preoccupied attachment status. When imaging proximity to a caregiver, these children demonstrated higher PNS reactivity which plays an inhibitory role and supports restoration while facilitating social engagement in low-threat environments. Furthermore, they showed a greater vagal withdrawal [lower respiratory sinus arrhythmia (RSA)] indicating a lower physiological arousal when experiencing the need for comfort from their caregivers. Another factor of importance might lie in temperamental traits and personality patterns. Psychoneuroendocrine stress research highlighted the interplay between personality and stress responses (Lecic-Tosevski et al., 2011). Future studies should also co-assess temperamental traits to further resolve interpersonal differences in *AAP*-related physiological reactivity.

Our findings raise some important questions concerning the physiological arousal in the four attachment groups. In line with previous results in infants (Spangler and Grossmann, 1993) and adults (Beijersbergen et al., 2008; Reijman et al., 2017) we found no significant differences in HRV between the group “unresolved” and the group “Ds.” It can be assumed that even though the unresolved attachment status precludes the presence of any coherent strategy, these individuals might employ passive and disengaging coping strategies to deal with attachment-related distress that are similar to those displayed in insecure individuals (Reijman et al., 2017; White et al., 2020). However, other research findings report differences between resolved and unresolved groups when using other attachment-related paradigms like the Cyberball experiment (De Rubeis et al., 2016). Thus attachment dysregulation might be very brief in an attachment interview (Beijersbergen et al., 2008) and it is not yet clear how much intensity is needed to observe attachment dysregulation on a physiological level (Beijersbergen et al., 2008; Gander and Buchheim, 2015). In this regard, the use of other measurements designed to assess attachment representations in middle childhood and adolescence might elicit different physiological responses. For example, the Family and Friends Interview (FFI, Steele and Steele, 2005) is theoretically based on the AAI but was adapted to the developmental abilities of children and adolescents between 8 and 16 years. The FFI differs from the AAI by considering a child’s reality and experiences in the context of friendships, siblings, self-perception and reflective functioning dimensions (Pace, 2014; Pace et al., 2020; Psouni et al., 2020). As the FFI inquiries about the adolescents’ view of the conflicting emotions arising in their close relationships rather than comparing semantic and episodic memories of past experiences with attachment figures like in the AAI (Pace, 2014), this technique might cause a higher emotional intensity and thus reveal different physiological patterns in those with a resolved and an unsolved attachment status. Another important aspect is that we did not subdivide the AAP into subphases with standardized time intervals. Although this is in line with other studies assessing physiological parameters during attachment interviews (i.e., Beijersbergen et al., 2008; Farina et al., 2015), a comparison of HRV estimates for certain subphases might be helpful to further justify deriving average scores between individuals with different attachment patterns. Furthermore, recent developments in virtual reality (VR) technology provided the applicability of this technological platform to confront study participants with an environmental stressor, the *Trier Social Stress Test* (TSST) being a prominent example for virtually experienced stress (Kirschbaum et al., 1993). To test physiological reactivity in the attachment context, future research might also use a virtual version of the *AAP*.

Furthermore, sample characteristics might be a reason for the inconsistent findings reported in unresolved individuals. Some studies only include clinical patients (Farina et al., 2015; De Rubeis et al., 2016), others focus on a very selective cohort of adolescents in foster care who reported experiences of postadoption loss and psychological trauma (Beijersbergen et al., 2008) and one study reports results from maltreating and non-maltreating mothers (Reijman et al., 2017). In our study, the group “unresolved” demonstrated the highest psychological distress level as measured with the *BSCL*. We hypothesize that unresolved individuals with a high-risk status display more dysregulation when confronted with attachment-related stimuli than those with a low-risk status which might result in a different physiological reactivity pattern. Hence, future research comparing clinical and non-clinical populations might provide an interesting insight into the complexity of the physiological arousal in individuals with an unresolved attachment status. In this regard, some researchers have proposed to further distinguish between two distinct neurobiological phenotypes of attachment disorganization deriving from attachment figures serving either as an insufficient or a threatening source of co-regulation (White et al., 2020).

Yet interestingly, our results did not show statistically significant differences in HR among the four attachment groups. Whereas HR provides information about cardiovascular activity – that is the average of the heartbeats per minute, HRV assesses specific changes in variability between successive heartbeats indicating the ability of the heart to respond to a variety of stress-related stimuli. Although our results on HR were not significant, we found a higher HRV in secure adolescents indicating that their stress-response system has a better ability to tolerate attachment-related distress. In line with other researchers who used HRV instead of HR to assess stress reactivity (Beijersbergen et al., 2008; Bryant and Hutanamon, 2018), our findings support the idea of HRV to be a viable measurement to enhance our understanding of an individual’s physiological regulation in response to attachment-related stress.

Despite the given strengths of the present study, we also had to deal with several limitations. First, although our sample size is comparable to the majority of studies on physiological correlates of attachment in adults (see for example Roisman et al., 2004; Farina et al., 2015; Reijman et al., 2017) and infants (see for example Spangler and Grossmann, 1993; Willemsen-Swinkels et al., 2000), a larger sample size might have provided sufficient statistical power to detect further differences on HRR between the four attachment groups. Second, we only included adolescents from the community. Studies comparing non-clinical adolescents to a high-risk or a clinical group of adolescents might shed light on possible links between autonomic regulation and attachment representations in individuals with different levels of caregiving quality. Third, we only distinguished between attachment categories but did not consider other attachment-related aspects like for example specific markers of dysregulation (Buchheim and George, 2011; Buchheim et al., 2018) that might play a key role in understanding the physiological arousal in response to attachment-related stressors. In this context, physiological research on attachment might also benefit from including information about chronic and traumatic life events like child abuse and neglect [i.e., by using the *childhood trauma questionnaire* (CTQ) or the *maltreatment and abuse chronicity of exposure* scale (MACE)] to gain a deeper understanding of an individual’s history of stress burden among the different attachment groups. A fourth limitation of the present study is the high number of participants (*n* = 23) who were excluded for technical problems during the physiological measurements. As we did not directly monitor raw data signals during the recordings, some datasets were lost mainly in the first third of the experiments. In future studies, it is necessary to better control for signal intensity and quality throughout the whole experimental procedure. Finally, some researchers view RMSSD as an accurate estimate of parasympathetic activity (Wulsin et al., 2018) whereas others view indices of parasympathetic activity derived from spectral analyses, the peak-to-valley or other time-domain methods as superior (Allen et al., 2007). Future work should therefore try to replicate the results using these methods which, at least in part, can be readily derived from freely available software packages, such as Kubios or Artifact.

The present study demonstrates that secure adolescents showed a higher HRV suggesting that they are more capable of dealing with attachment-related stressors. HRV is increasingly recognized as a marker of feeling safe and connected in social environments (Bryant and Hutanamon, 2018) and thus our results might have important implications for psychotherapy research. Studies testing the efficacy of novel attachment-based treatments that foster the security in attachment-relationships in adolescence (Kobak and Kerig, 2015) might benefit from including HRV as a primary outcome variable (Kirby et al., 2017).

Interdisciplinary research allows the continuously increasing process of integrating psychological and biological measures into attachment research. Future studies integrating biomarkers of attachment, e.g., oxytocin and prolactin, that can be measured in different body fluids (i.e., saliva, urine, and blood serum) might be helpful to further investigate the physiological and biomolecular underpinnings of attachment-related stress responses.

Additionally to such hypothesis-driven selections of biomarker candidates, non-targeted omics-analyses (e.g., metabolomics, proteomics, and lipidomics) can be used to identify the complex biology of different attachment representations as well as their differences in the physiological responses to the *AAP*.

## Data Availability Statement

The raw data supporting the conclusions of this article will be made available by the authors, without undue reservation.

## Ethics Statement

The studies involving human participants were reviewed and approved by University of Innsbruck. Written informed consent to participate in this study was provided by the participants’ legal guardian/next of kin.

## Author Contributions

MG significant contribution to the conception of the study, coding of AAP interviews and contribution to the data analysis regarding attachment, responsible for writing the manuscript, and revising the work for its’ intellectual content. AK significant contribution the data analysis regarding physiological measurements, responsible for writing parts of the manuscript (measurement and discussion section), and revising the work for its’ intellectual content. KN contributed to the data analysis and interpretation regarding physiological measurements and responsible for writing parts of the manuscript (measurement section). DB responsible for AAP codings and interpretation of the data, contributed to writing parts of the manuscript (AAP measurement), and revising the work for its intellectual content. CD-W responsible for coding of attachment interviews and interpretation of the data regarding attachment measurement. AB major contribution to the study design and interpretation of the data and responsible for writing parts of the manuscript (discussion and introduction section). All authors contributed to the article and approved the submitted version.

## Conflict of Interest

The authors declare that the research was conducted in the absence of any commercial or financial relationships that could be construed as a potential conflict of interest.

## Publisher’s Note

All claims expressed in this article are solely those of the authors and do not necessarily represent those of their affiliated organizations, or those of the publisher, the editors and the reviewers. Any product that may be evaluated in this article, or claim that may be made by its manufacturer, is not guaranteed or endorsed by the publisher.

## Abbreviations

AAP: *Adult Attachment Projective Picture System*
AAI: *Adult Attachment Interview*
BLQ: *baseline questions*
BLR: baseline relaxation/silence
BSCL: *Brief Symptom Checklist*
Ds: insecure-dismissing
E: insecure-preoccupied
F: secure
FFI: Family and Friends Interview
HR: heart rate
HRR: heart rate reactivity
HRV: heart rate variability
RMSSD: root mean square of successive differences
SDNN: standard deviation of NN intervals
U: unresolved.
